# Association mapping of QTLs for sclerotinia stem rot resistance in a collection of soybean plant introductions using a genotyping by sequencing (GBS) approach

**DOI:** 10.1186/s12870-014-0408-y

**Published:** 2015-01-17

**Authors:** Elmer Iquira, Sonah Humira, Belzile François

**Affiliations:** Département de Phytologie and Institut de biologie intégrative et des systèmes, Université Laval, Quebec City, Quebec Canada

**Keywords:** Soybean, Sclerotinia, QTL, Association mapping

## Abstract

**Background:**

Sclerotinia stem rot (SSR) is the most important soybean disease in Eastern Canada. The development of resistant cultivars represents the most cost-effective means of limiting the impact of this disease. In view of ensuring durable resistance, it is imperative to identify germplasm harbouring different resistance loci and to provide breeders with closely linked molecular markers to facilitate breeding. With this end in view, we assessed resistance using a highly reproducible artificial inoculation method on a diverse collection of 101 soybean lines, mostly composed of plant introductions (PIs) and some of which had previously been reported to be resistant to sclerotinia stem rot.

**Results:**

Overall, 50% of the lines exhibited a level of resistance equal to or better than the resistant checks among elite material. Of the 50 lines previously reported to be resistant, only 20 were in this category and a few were highly susceptible under these inoculation conditions. The collection of lines was genetically characterized using a genotyping by sequencing (GBS) protocol that we have optimized for soybean. A total of 8,397 single nucleotide polymorphisms (SNPs) were obtained and used to perform an association analysis for SSR by using a mixed linear model as implemented in the TASSEL software. Three genomic regions were found to exhibit a significant association at a stringent threshold (*q* = 0.10) and all of the most highly resistant PIs shared the same alleles at these three QTLs. The strongest association was found on chromosome Gm03 (*P*-value = 2.03 × 10^−6^). The other significantly associated markers were found on chromosomes Gm08 and Gm20 with *P*-values <10^−5^.

**Conclusion:**

This work will facilitate breeding efforts for increased resistance to Sclerotinia stem rot through the use of these PIs.

**Electronic supplementary material:**

The online version of this article (doi:10.1186/s12870-014-0408-y) contains supplementary material, which is available to authorized users.

## Background

White mold on soybean, also known as Sclerotinia stem rot (SSR), is an important disease in the northern USA, Argentina, China and regions of Canada where soybeans are grown [1,2]. In the United States, SSR is considered to have been the second most important cause of soybean yield loss in 1994 [3], in 2004 [4] and 2009 [5]. In Canada, it was also the second most important disease on the soybean crop in 1994. In 1996, SSR caused 20% yield losses in Quebec [6] and it has been considered the most important disease for soybean production in this part of Canada for the last 20 years.

Breeding for SSR resistance is difficult, as this resistance is controlled by multiple genes [7-11]. Screening for resistance is also challenging because infection and disease development in field plots is often inconsistent. Recently, however, we have developed a simple and reliable inoculation method wherein mycelium is applied to floral buds [12]. The resulting lesions progress more or less rapidly along the main stem according to the resistance offered by each genotype. Because of its simplicity and reproducibility, it is well suited to characterize new germplasm and for QTL mapping studies. We have already used this method to map reproducible QTLs conferring SSR resistance in the Canadian cultivar Maple Donovan [13] and among a panel of elite Canadian cultivars [14].

Resistance has also been reported in a large number of plant introductions using a range of inoculation methods [9]. Unfortunately, very little work has been done to characterize the genetic architecture of the resistance in these PIs. It would be important for breeders to know if these PIs contain additional or different QTLs conferring resistance to SSR, relative to those found in elite material, in view of designing adequate crosses and selection strategies to introduce such beneficial alleles. For these purposes, mapping is an attractive strategy to rapidly identify QTLs in a collection of PIs.

Association analysis is based on linkage disequilibrium (LD) and complements conventional linkage mapping for the identification of genes and QTLs for traits of interest. This new approach has been receiving unprecedented attention because of its advantages, including high resolution, cost efficiency, and non-requirement of pedigrees or crosses. Moreover, genome-wide association studies (GWAS) are useful and powerful for the identification of the genetic variations that underlie many important and complex phenotypes such as disease resistance. Hence, GWAS can reduce costs and time for genetic dissection of traits. This approach has been used in soybean to identify genes associated with iron deficiency chlorosis [15]; chlorophyll content and chlorophyll fluorescence parameters [16], yield and yield components [17], SSR resistance in a collection of elite soybean lines [14] and seed protein and oil content [18]. All but the last of these studies used the Universal Soybean Linkage Panel [19], a Golden Gate assay that allows one to interrogate 1,536 SNPs at a time, a subset of which will be informative in a given set of materials [20]. According to Hyten et al. [21], as well as more recent studies [14,18], tens of thousands of SNP markers would be required to exhaustively cover the genome for the purpose of genome-wide genetic analysis. Therefore, SNP genotyping platforms capable of determining the genotypes at a larger number of SNP loci are required to perform more powerful association studies in soybean.

Alternatively, the genotyping by sequencing (GBS) approach in plants has been recently developed [22], and was adapted to soybean [23] offering a very versatile genotyping technology with a very good resolution and low cost. We initiated a genome-wide association study using GBS-derived SNPs in order to identify the chromosomal regions associated with SSR resistance. With this in view, a population of 101 soybean genotypes was assessed for their level of resistance against sclerotinia infection. The aim of the present work was to identify the gene(s) or QTL(s) that significantly affect soybean white mold resistance.

## Results

### Reaction to SSR inoculation

**Figure 1:**
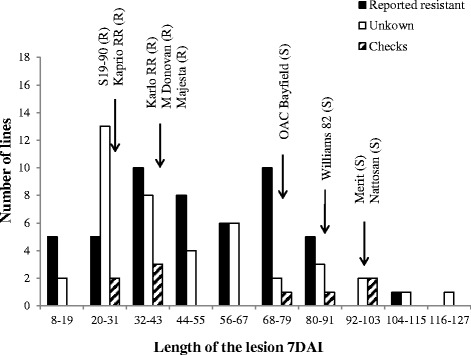
**Phenotypic distribution of Sclerotinia stem rot lesion length in 101 soybean genotypes after inoculation with the cotton pad method.** Progression of the lesions was assessed 7 d after inoculation at R1. The soybean lines are separated in three subgroups: 1) previously characterized as resistant [9] (black), 2) previously uncharacterized for resistance to *S. sclerotiorum* (white), or 3) resistant (R) and susceptible (S) checks (striped).

The panel of 101 genotypes (including both resistant and susceptible checks) were inoculated with the cotton pad method in greenhouse conditions to characterize their phenotypic response to SSR infection. Under controlled conditions in which very high humidity could be maintained, the mean length of lesions covered a broad range, from as little as 13 mm to as long as 124 mm, with a population-wide average of 51 mm (Additional file 1: Table S1 and Figure 1). All four resistant checks exhibited mean lesion lengths from 23 to 37 mm, while the four susceptible checks had lesions averaging more than 78 mm, with the most susceptible check (Nattosan) having lesions averaging 102 mm. It is noteworthy to mention that all accessions developed a lesion; even the most resistant genotype developed a short lesion indicative that it was infected but was able to stop the development of the fungus.

Half of the lines (50%) exhibited a very good level of resistance (equal to or better than our resistant checks), 40% showed intermediate resistance (between our resistant and susceptible checks) and 10% were highly susceptible (more susceptible than our susceptible checks). Of the subset of 50 accessions that had been previously reported as resistant, only 20 were at least as resistant as the resistant checks used in this work. Among this subset, five accessions (PI391589B, PI507352, PI561345, PI196157 and PI398637) were the most resistant genotypes of all, even more resistant than the most resistant check (S19-90). A second group of five accessions (PI358318A, PI189919, PI189861, PI437527 and PI549066) were similar to S19-90 and a third group of 10 accessions (PI567157A, PI416776, PI561331, PI437764, PI507353, PI548312, PI504502, PI437072, PI89001 and PI243547) performed as well as the remaining resistant checks. Again among this subset previously reported as resistant, a group of 14 accessions developed lesions of intermediate length and a final group of 16 accessions developed lesions equal or longer than the susceptible checks. Overall, the results of the cotton pad method were not significantly correlated with the DSI ratings reported by Hoffman et al. [9] (data not shown).

In the remaining subset composed of 42 lines of unknown reaction to SSR, 23 accessions performed as well as the resistant checks. Of these, two genotypes (PI423949 and PI603148) were more resistant than S19-90 and another 13 accessions were equally resistant as S19-90. Another group of eight accessions (PI593973, PI281850, PI423941, PI194634, PI503336, PI424242, PI593972 and PI458520) performed as well as the remaining resistant checks. Finally, among this subset, ten lines showed an intermediate response and nine accessions were at least as susceptible as the susceptible checks.

### SNP discovery and distribution

**Figure 2:**
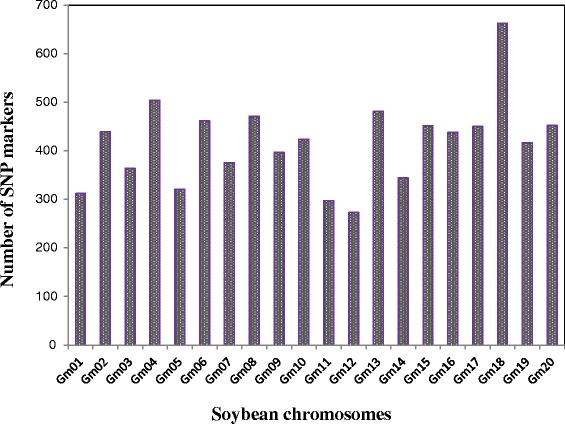
**Distribution of SNP markers on the 20 soybean chromosomes.**

A total of 145,347 “raw” SNPs and InDels were identified using the IGST-GBS [23] variant calling pipeline. After strict filtering of SNPs on the basis of read depth and minor allele frequency (MAF > 0.05), a final set of 8,397 SNPs (InDels were not used) was obtained and used for association analysis. Of these 8,397 SNPs, 8,339 map to assembled chromosomes while the remaining markers (58) mapped to scaffolds that remain unassigned to a chromosome. These 8,339 SNP markers were distributed over all 20 chromosomes with a median distance between markers of 32 kb and an average of 416 SNP markers per chromosome (Figure 2). The greatest number of SNPs was detected on chromosome 18 (663 SNPs), followed by chromosome 4 (504 SNPs) and the lowest was observed on chromosomes 12 (273 SNPs) and 11 (297 SNPs).

### Population structure and linkage disequilibrium

**Figure 3:**
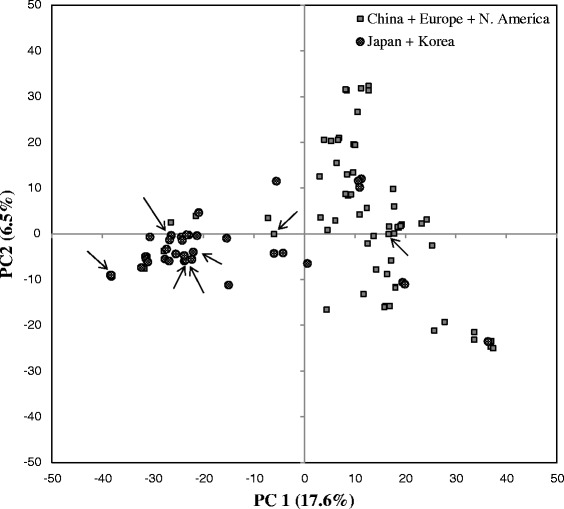
**Principal component analysis.** The PC analysis of 101 soybean lines was conducted using 2,593 SNP markers with MAF ≥ 0.3. The two-dimensional plot (PC1 vs PC2) shows that lines are assigned to two main groups according to their geographical origin. Arrows indicate the seven most resistant accessions in the whole panel.

The genetic structure of the 101 soybean lines was explored by PCA using a subset of 2,593 SNP markers (with MAF ≥ 0.3). In this population, a total 29.4% of the variance was explained by the first three principal components (17.6, 6.5, and 5.0% respectively). The two-dimensional scatter plot (PC1 vs PC2) involving all accessions displayed two main subpopulations (Figure 3). It divided the accessions into the lines coming from China and those coming from Japan and Korea. Most of the European accessions were grouped with the Chinese lines as were the North American cultivars. The seven most resistant accessions did not cluster together but rather were distributed according to their geographical origin.

**Figure 4:**
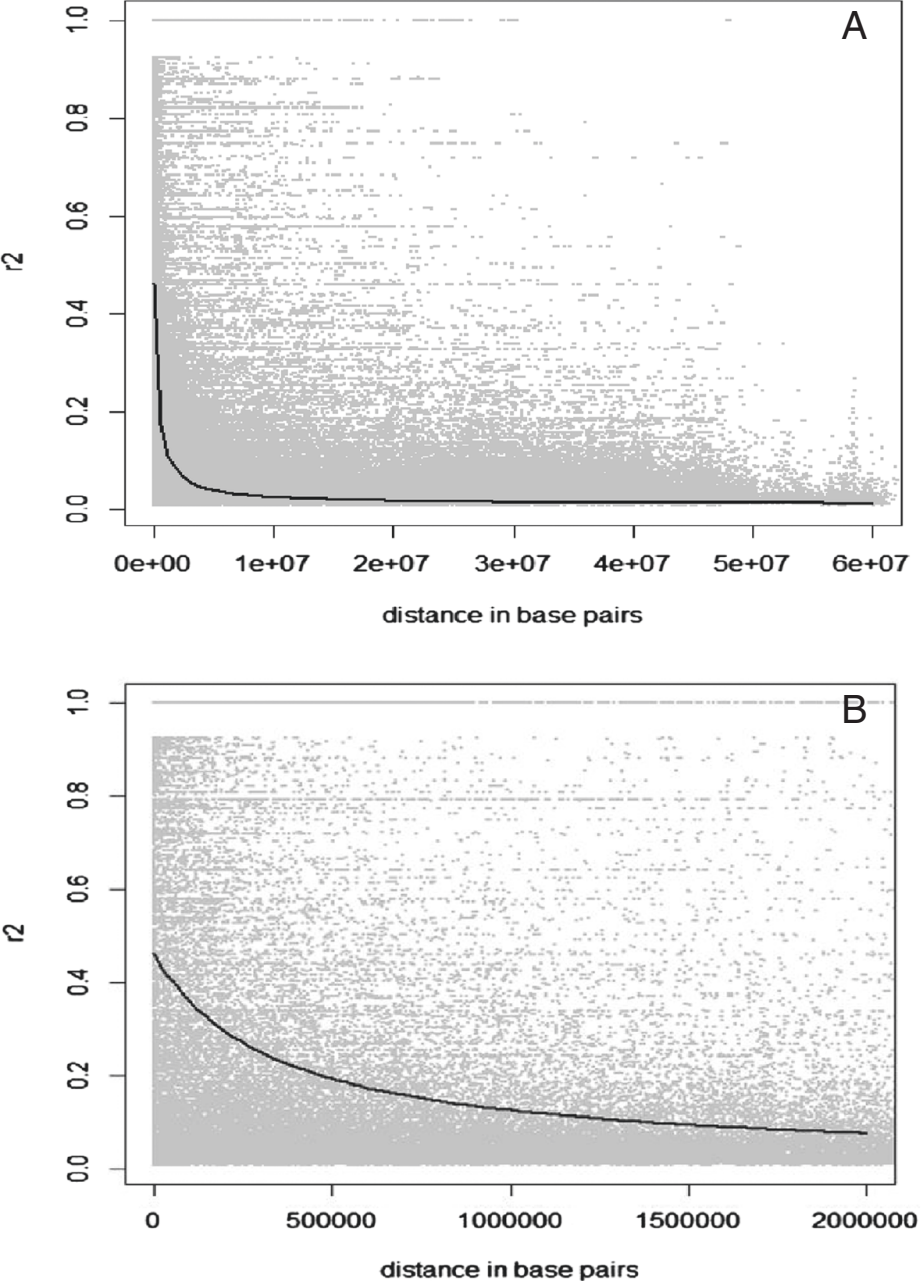
**Intra-chromosomal LD (r** ^**2**^ **) decay among marker pairs over all chromosomes as a function of physical distance (bp). A)** Panoramic view; **B)** More detailed view of LD decay within 2Mb.

The intra-chromosomal LD was calculated and pairwise r^2^ was calculated for all SNPs across the soybean genome. Only significant r^2^ values (*P* < 0.001) were considered as informative. Among all loci pairs (1,807,882), only 11.8% were in significant LD in the whole panel. Significant intra-chromosomal r^2^ values ranged from 0.09 to 1 with an average of 0.28. Out of all loci pairs in significant LD, 51.7% of these had an r^2^ value above 0.2, 12.5% had an r^2^ value above 0.5 and only 2.1% were in complete LD (r^2^ = 1). The decay of LD with increasing physical distance is illustrated in Figure 4a. On average, intra-chromosomal LD declined below r^2^ = 0.2 at around 500 kb (Figure 4b).

### Genome–wide association analysis

**Figure 5:**
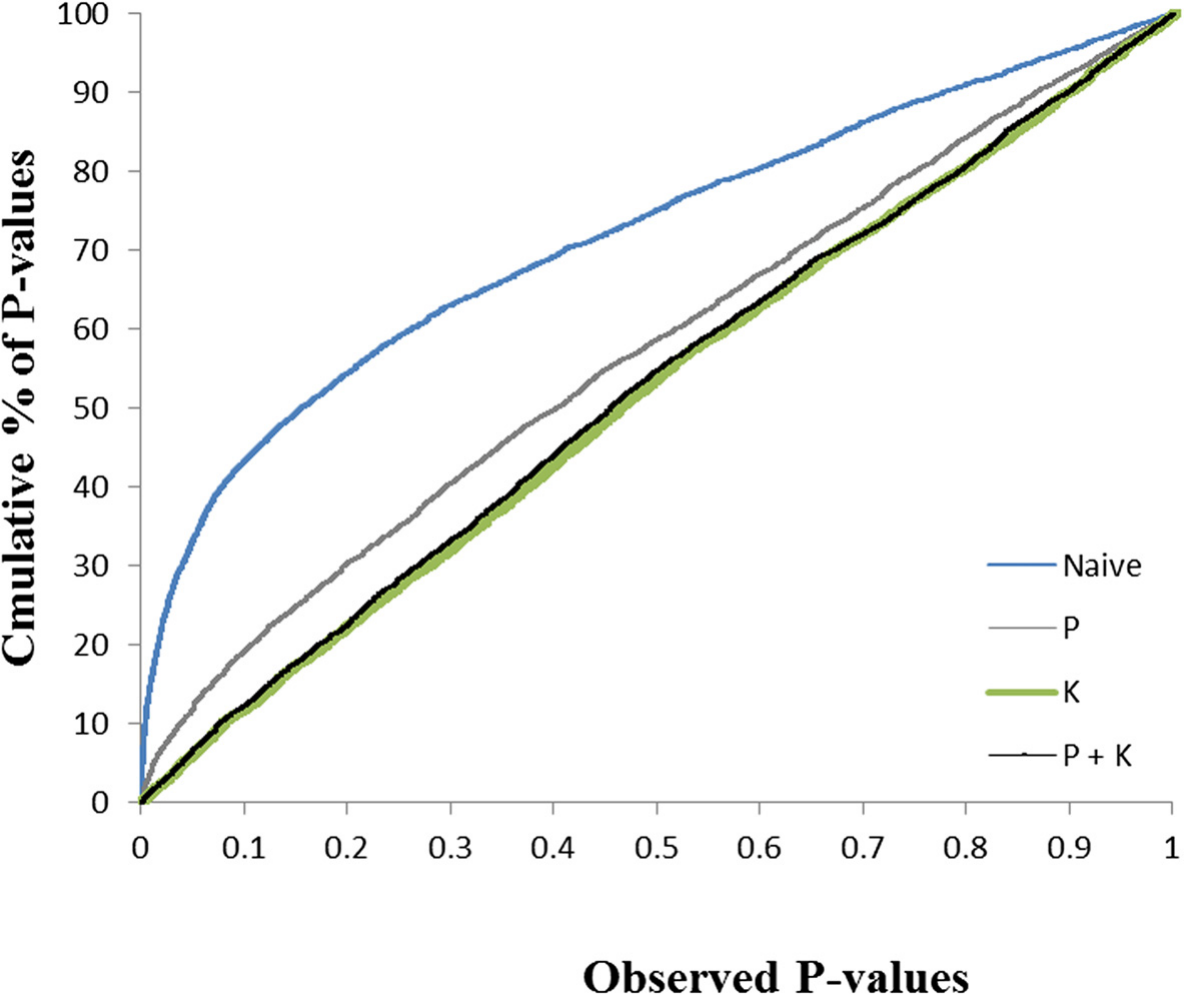
**Comparison of four genome-wide association models.** Cumulative distribution of *P*-values computed from 8,397 SNPs for the naïve model, P model, K model and the K + P model.

We tested four models to detect associations between SNP markers and SSR resistance, a trait that exhibited a heritability of 67%. As expected and illustrated in Figure 5, a large proportion (33.3%) of marker-trait associations showed *P*-values < 0.05 when using the naive model that does not take into account population structure and genetic relatedness. Using a model accounting only for population structure (model P), the proportion of *P*-values < 0.05 decreased to 12.1%, but still suggested a large number of false positive associations. In contrast, the K and P + K models both showed a much improved fit between observed and expected *P*-values, with only 6.2% and 6.8% of *P*-values < 0.05, respectively. Accordingly, the cumulative distribution of *P*-values largely followed a diagonal. This result suggests that mixed linear models using either K alone or P + K accounted very well for population structure and genetic relatedness among these lines.

**Figure 6:**
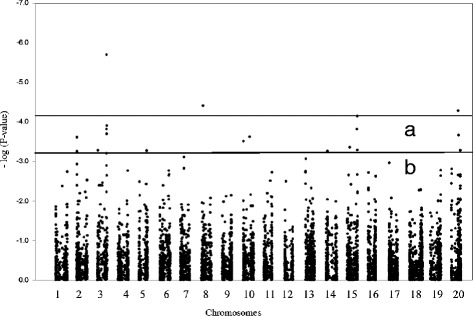
**Genome-wide association scan for** ***Sclerotinia*** **lesion length.** The soybean genome was scanned with a total of 8,397 SNPs using a mixed linear model (P + K) taking into account both population structure and genetic relatedness. The vertical axis plots the –log10(*P*) values of the association between the markers and lesion length. The horizontal line shows the threshold above which *P*-values correspond to a false discovery rate of < 0.10 **(a)** and < 0.20 **(b)**.

To account for the large number of markers being tested and to set a reasonable false discovery rate, a *q*-value was calculated for the whole set of *P*-values. A threshold *q*-value equal to 0.10 was chosen and corresponded to *P*-values < 7 × 10^−5^. At this significance level, 4 SNP markers located in 3 genomic regions (on chromosomes Gm03, 08 and 20) met this stringent criterion (Table 1 and Figure 6). On Gm03 and 20, a single SNP marker exceeded this threshold, although many neighbouring markers often showed *P*-values < 0.0001. On Gm08, two SNP markers (44 kb apart) were both equally tightly associated with SSR resistance (*q*-value = 0.10).

**Table 1:** **SNP markers strongly associated with the length of the lesion after the confrontation with** *Sclerotinia sclerotiorum*

**Chrom.**	**SNP position (bp)**	***p*** **-value**	***q*** **-value**	**R2 (%)**	**MAF**	**Minor allele mean**	**Major allele mean**
3	44,735,630	2.03^E-06^	0.01	21	0.14	79.4	46.7
8	7,606,596	3.91^E-05^	0.10	16	0.06	101	48.1
8	7,650,317	3.91^E-05^	0.10	16	0.06	101	48.1
20	33,511,401	5.30^E-05^	0.10	15	0.4	64.3	42.6
15	47,443,434	7.30^E-05^	0.11	12	0.47	80,5	33,5
2	2,385,261	2.44^E-04^	0.14	13	0.08	83.9	48.5
10	31,766,279	2.42^E-04^	0.14	13	0.06	93.5	48.6
10	2,829,577	3.12^E-04^	0.16	13	0.38	57	48
3	3,012,147	5.26^E-04^	0.17	12	0.09	87.2	47.8
14	4,612,686	5.44^E-04^	0.17	12	0.23	71.4	45.3
15	12,233,432	4.40^E-04^	0.17	12	0.06	99.8	48.2
20	42,688,433	5.30^E-04^	0.17	12	0.32	71.5	41.9

The most significant association was found with a SNP marker on Gm03 (*P*-value = 2 × 10^−6^; *q*-value = 0.01). This marker alone accounted for 21% of the phenotypic variation in this population and lines carrying the resistance allele at this marker had lesions that were 32.7 mm shorter on average than lines with the alternate allele. The two other genomic regions (Gm08 and Gm20) shared a very similar degree of association with SSR resistance (*q*-value of 0.10) and accounted for a similar amount of phenotypic variance (15-16%). The allelic effect of these other SNP markers ranged between 21.7 mm (Gm20) and 52.9 mm (Gm08). In all cases, the most frequent allele was favourable as it was associated with shorter lesions.

To widen the scope of the search, a more permissive critical *q*-value (0.2) was used and 8 additional marker-trait associations with *P*-values < 7 × 10^−4^ were found to be significant at this second threshold (Table 1). The *q*-values for this second tier ranged between 0.11 and 0.17, with each marker accounting for 12-13% of the phenotypic variation. Allelic effects at these marker loci ranged between 9 and 51.6 mm. Globally, the variance explained by all of the 12 significant markers was estimated to be 41%.

**Figure 7:**
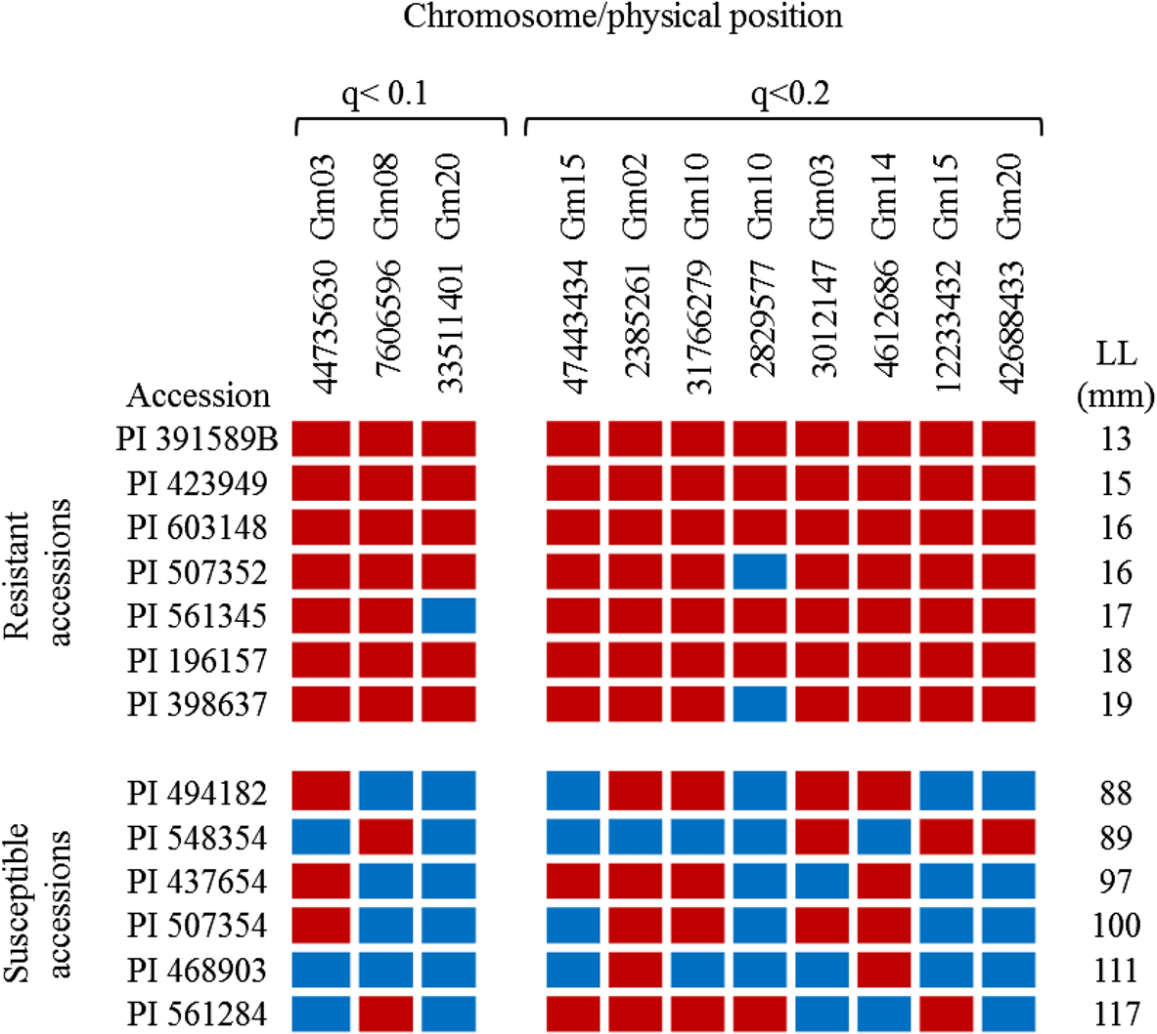
**Genotype of the seven most resistant (upper block) and six most susceptible (lower block) accessions at the putative QTLs identified using a q-value < 0.2.**

Finally, we examined the three chromosomal regions harbouring significant marker-trait associations in order to see how alleles at these loci were distributed in the most resistant and susceptible accessions. As can be seen in Figure 7, with a single exception, the seven most resistant accessions were fixed for the resistance allele at all three QTLs. For these same loci, the six most susceptible lines carried mostly, but not exclusively, the alleles associated with increased lesion length. Similarly, among the next tier of QTLs (0.1 < *q* < 0.2), a strong predominance of resistance alleles (54 out of 56 alleles) was observed whereas among the most susceptible lines, 26 unfavourable alleles were found (out of a total of 48). As it was the case for the first exercise, resistance alleles were mostly present for these markers. However, the allelic portrait for the most susceptible accessions is much more variable, they show a mixture of susceptible and resistant alleles, but with a big proportion of susceptible alleles.

## Discussion

### Reaction to SSR inoculation

In this study, SSR resistance was assessed in a collection of 101 soybean PIs and cultivars composed of a first subset of 50 lines previously reported to be resistant [9], a second subset of 42 lines that had not been previously tested and 9 checks. The cotton pad method allowed us to rate the accessions based on their ability to halt or slow the progression of the pathogen. Twenty of the 50 accessions reported as resistant to SSR performed as well as the resistant checks and five of these were found to be even more resistant than the best resistant check (S19-90). The remaining lines were found to be tolerant, moderately tolerant or even susceptible to the spread of SSR. Therefore, our results are not in agreement with the previous report [9]. Such a discrepancy may be due to differences in the assays used to assess disease resistance. Whereas our method assesses a specific component of resistance, i.e. those mechanisms contributing to restrict pathogen spread once inside the plant, the disease severity index used by Hoffman et al. [9] provides a broader measure of resistance (Additional file 2: Table S2). For example, flowering time or plant architecture (avoidance mechanisms) could contribute to the resistance as measured by Hoffman et al. [9], but cannot in our tests as the pathogen is put in direct contact with the flowers.

Interestingly, in the other subset of soybean lines, two additional accessions (PI423949 and PI603148) performed better than the most resistant checks, thereby contributing to the list of accessions providing a high degree of resistance to pathogen spread. These genotypes had not been reported before as sources of resistance to SSR. This finding suggests that there could be more useful sources of resistance in the soybean germplasm.

Interestingly, accession PI423949 was reported to be a source of some race-specific resistance for *Phytophthora sojae* [24]. Other soybean lines reported resistant to some races of *P. sojae* [25] also showed a good tolerance to SSR such as accession PI196157 (one of the five accessions identified as the most resistant in the first subset). Because these pathogens are genetically unrelated and infect different plant tissues, this apparent dual resistance might be conferred by genes related to general defense responses. Alternatively, it could also be a mere coincidence that these genotypes exhibit resistance to both diseases. It would be interesting to further explore the possible relationship between resistances to the spread of these two fungal pathogens.

### Number of SNPs and genome coverage

In this work, a set of 8,397 SNPs was obtained using a GBS approach to genotype the collection of soybean lines. In recently published work in soybean, association analyses have been used to investigate the association of SNPs with SSR resistance [14], iron deficiency chlorosis [15], chlorophyll and chlorophyll fluorescence parameters [16], yield and yield components [17], as well as seed protein and oil content [18]. Most of these studies used a relatively small number of markers ranging from 850 to 1,142 markers. Thus, the number of SNPs used here was 8- to 10-fold greater than used in all but the most recent association analyses in soybean. Among the latter, one study relied on ~8,000 SNP markers [14] while the most recent study [18] examined close to 32,000 SNPs. Although the number of markers examined here undoubtedly contributed to a more extensive genome coverage, it still falls somewhat short of the number of informative tag SNPs that are thought to be needed to capture most of the haplotypes within the euchromatic regions of the soybean genome; this number has recently been estimated to be around 60,000 markers, although this would be affected by the composition of the association panel [14]. Hence, our study may have failed to detect some QTL because of insufficiently dense marker coverage.

### Population structure and linkage disequilibrium

Principal component analysis indicated that this collection had two distinguishable subpopulations. The first subpopulation was comprised mostly of Chinese accessions and these were separated from the second subgroup formed mostly of Japanese and Korean accessions. Such groups reflecting the geographical origin of soybean accessions within different regions of Asia have been documented previously in numerous studies of genetic diversity [26-28].

In our collection of soybean lines, LD extended to 500 kb on average over the entire genome. Such extensive LD in soybean has also been reported in other studies assessing genome-wide LD [17,29-31], although all of these were conducted with a much smaller number of markers, as noted above. Our finding is also consistent with a previous report [19] in which LD was measured on a local level using a high density of SNPs (ranging from 1 SNP/12.4 kb to 1 SNP/57.4 kb). In the latter study, LD was found to extend from 90 to 574 kb among cultivated soybean. As LD in the largely heterochromatic pericentromeric regions is generally more extensive, one would predict that LD within the euchromatic regions would be smaller and on the order of what was reported earlier [19]. Finally, our results are consistent with a resequencing study [28] where LD decayed to half of its maximum value at 150 kb for cultivated soybeans.

### Genome –wide association of resistance to Sclerotinia stem rot

Sclerotinia stem rot is a necrotrophic fungus and it has a broad range of host species including soybean. There is no complete resistance to sclerotinia in soybean because the resistance is quantitatively controlled by numerous genes or quantitative trait loci (QTLs). More than 30 QTLs responsible for SSR have been reported in soybean [7,8,10,13,14,32]. However, all but one [14] of these studies have been limited to conventional biparental mapping populations from a small number of parents, which limits the alleles segregating in the progeny to those that differ in the parental lines.

From our diverse mapping panel including 101 lines, we identified 4 SNP markers located in 3 genomic regions (on chromosomes 3, 8 and 20) showing significant association with disease resistance at a stringent threshold (*q*-value < 0.10). Together, these three genomic regions explained 41% of the phenotypic variance. As the size of our association panel was small, we expect to capture only large effect QTL and some additional QTLs may have eluded detection. Of these three regions, the one on Gm20 (peak SNP at 33.5 Mb) is very close (~70 kb) to a SSR marker (Satt354 at 33.4 Mb) previously found to be associated with SSR resistance using this same inoculation technique [13]. As for the other two chromosomal regions most highly associated with resistance (on Gm03 and Gm08), these do not coincide with previously reported QTLs for SSR resistance.

At a less stringent threshold (*q* < 0.20), 8 additional SNP markers located on chromosomes 2, 3, 10, 14, 15 and 20 were identified. One of these (Gm14 at 4.6 Mb) lies within a large interval (from 0.67 to 4.94 Mb) delimited by two SSR markers (Satt577 and Satt126) and reported by Vuong et al. [32] to harbour a QTL for SSR resistance.

The fact that we used a different set of accessions and inoculation method relative to previous work in this area may explain this lack of overlap. Other reasons explaining the inconsistency of estimated QTL effects could as well include i) genome coverage was not equally similar, ii) the QTL segregating in different mapping populations were also different, iii) a QTL x genetic background interaction was observed, and (iv) a probable QTL x environment interaction.

Finally, we compared our results with those found by GWAS in a population of Canadian soybean cultivars [14]. This population was inoculated with the pathogen by the same method and was genotyped with a similar number of SNPs. None of the QTLs identified in these two association studies proved to be the same. One explanation for this unexpected result is that the two populations had a different genetic background. A PCA showed that the collection of PIs studied here and the panel of elite germplasm studied by Bastien et al. [14] formed highly differentiated populations (Additional file 3: Figure S1). It is also important to note that in our population the resistance alleles for the most significant markers (Figure 7) seemed to be fixed in the seven most resistant accessions, in contrast with the situation observed in the Canadian soybean panel where the resistance alleles were not all present in the most resistant soybean cultivars. This observation matches very well with the fact that, in our panel, the proportion of resistant genotypes was more important than in the elite panel. Therefore, resistance alleles would be overrepresented in our panel.

### Practical implications for breeding

The seven most resistant soybean accessions identified in our study are interesting for use in any soybean breeding program. Although these accessions constitute different sources of resistance, these genotypes have mostly the same resistance alleles for the most significant SNP markers. So, breeders could choose one or more than one accession to introduce this resistance in their program. Even if resistance exists in elite soybean, the resistance observed in these accessions leads us to think that they are harboring new QTLs. Furthermore, adaptation, maturity group and resistance genes for other diseases could also constitute criteria for choosing a resistant accession in a cross. Finally, the introduction of resistance alleles from these exotic lines into an elite soybean breeding program could be facilitated by using the SNP markers associated with white mold resistance, but it would be necessary to develop a large-scale assay for rapid, reliable, and cost effective SNP genotyping. Nevertheless, the overall resistance of accessions seems to be controlled by a relatively high number of loci. Due to this high number of loci involved, breeding for quantitative SSR resistance will probably require strategies capable of exploiting multiple QTLs such as genomic selection [33].

## Conclusions

We took advantage of a panel of soybean accessions to perform an association mapping study to discover loci associated with SSR resistance in soybean. The discovery that some of the SNP markers mapped near previously discovered disease resistance QTLs further substantiates that this approach is a valuable experimental method with potentially broad applications for soybean genetics and breeding. Further studies, perhaps using a linkage mapping approach, are needed to confirm whether the SNP markers are truly linked to previously undetected QTL for SSR resistance.

## Methods

### Plant material

A panel of 101 soybean genotypes was used. It was composed of 50 accessions previously reported to be partially resistant to SSR [9] and belonging to maturity groups 000 to III. A further 42 accessions not previously tested for their resistance to SSR but reported to be sources of resistance to other diseases [24,34] were also included in this collection. Finally, nine elite lines were used as checks as these were known to be resistant (R), or susceptible (S): Maple Donovan (R), Majesta (R), Karlo RR (R), Kaprio RR (R), S19-90 (R), Merit (S), Nattosan (S), OAC Bayfield (S) and Williams 82 (S) based on previous reports [32,35-37].

### Disease assessment

Soybean seeds were sown in 6 L-pots containing 50% black earth, 30% perlite and 20% Promix (Premier Tech Horticulture, Rivière-du-Loup, QC). The experimental unit consisted of three 6-L pots sowed with 4 seeds inoculated with *Bradyrhizobium japonicum* (RhizoStick, Ames, IA) at sowing. After germination, plants were thinned to two per pot and grown under natural light supplemented with 600 W high-pressure sodium lamps (P.L. Light Systems, Beamsville, ON) to provide a 16-h photoperiod. During growth prior to inoculation, the day/night temperatures were 26°C/20°C. Inoculations were performed on one young flower bud per plant when both plants had reached the R1 growth stage and were conducted using cotton pads drenched in a mycelial suspension as previously described by Bastien et al. [12]. Immediately after inoculation, plants were transferred to another greenhouse compartment where day/night temperatures were 24°C/18°C. Humidity was controlled based on water pressure deficit (maintained at 2.5 g m^−3^ with a fogging system). Lesion length was measured 7 days after inoculation. The experiment was a randomized complete block design with three replications separated by time during the winter 2009–2010. Planting dates were 30 October 2009, 8 December 2009, and 20 January 2010.

### DNA extraction, library preparation and sequencing

DNA was extracted from 100 mg fresh young leaves using the DNeasy 96 Plant kit (Qiagen, cat. no. 69181) following the manufacturer’s protocol. DNA was quantified using a Nanodrop 8000 spectrophotometer (Thermo Scientific, http://www.thermoscientific.com). DNA concentrations were normalized to 10 ng/μl and subsequently used for library preparation. Genotyping by sequencing libraries (96-plex) were prepared according to the *Ape*KI protocol described by Elshire et al. [22]. Single-end sequencing was performed on an Illumina HiSeq 2000 at the McGill University-Génome Québec Innovation Center in Montreal, Canada.

### Processing of Illumina raw sequence read data and SNP calling

Read processing, mapping and initial SNP calls were performed using the IGST-GBS pipeline described by Sonah et al. [23]. Raw SNPs were further filtered using VCFtools to retain SNPs with less than 20% missing data and a minor allele frequency greater than 0.05. Any heterozygous genotype calls were treated as missing data. Finally, missing genotypes were imputed using fastPHASE Version 1 [38].

### Statistical analyses

LD was calculated using TASSEL 3.0 [39] with default settings and pairwise r^2^ was calculated for all SNPs across each chromosome of the soybean genome. Only significant r^2^ values (*P* < 0.001) were considered as informative. LD decay was calculated using the method described by Remington et al. [40] in which a non-linear least squares estimate of r^2^ per base pair is estimated. To compute the expected values E (r^2^), the formula from Hill and Weir [41] was used in an R script (http://www.r-project.org/).

### Genome –wide association analysis

Four types of models, a general linear model (GLM) and mixed linear models (MLM), were selected to test marker-trait associations. Principal component analysis (PCA) was used to describe population structure and was performed in TASSEL 3.0 using 2,593 SNPs (MAF ≥ 0.3) and the first three significant PCs were used based on the resulting Scree plot. A kinship matrix was produced using TASSEL 3.0 with 8,397 SNP markers (MAF ≥ 0.05) to estimate genetic relatedness between the lines. The following models were tested: i) Naive model: GLM without any correction for population structure; ii) P-model: GLM with 3 PCs; iii) K-model: MLM with the K matrix; and iv) PK-model: MLM with 3 PCs and the K matrix. The critical *P*-values for assessing the significance of marker-trait associations were calculated based on their corresponding q-value. A q-value of 0.10 was used as a significant association threshold in addition to a more permissive threshold of 0.2. Considering that a q-value is a measure of significance in terms of the false discovery rate [42], we chose to use a cut-off of 0.1 because it is considered conservative for such marker discovery work that can be subject to further validation [43,44].

The heritability was calculated by using GAPIT software [45]. The GCTA software was also used to estimate the variance explained by the significant SNP markers [46].

### Availability of supporting data

The raw sequencing data for every sample has been deposited in NCBI-SRA and is accessible through the BioProject number PRJNA269246 (http://www.ncbi.nlm.nih.gov/bioproject/?term=PRJNA269246).

The phenotypic data, significant SNPs, list of all SNPs, etc. have been deposited in SoyBase and the accession number is SoyBase.C2014.01 (http://soybase.org/projects/SoyBase.C2014.01.php).

## Additional files

Additional file 1: Table S1. Details of the 101 soybean genotypes used for GWAS. Name of the accession, country of origin, maturity group and mean Sclerotinia stem rot lesion length (LL). Additional file 2: Table S2. Comparison for the mean Sclerotinia stem rot ratings for the field test according to Hoffman et al. [9] and the lesion length for the cotton pad inoculation method (Bastien et al. [12]) of soybean plant introductions and the check cultivar S19-90. Additional file 3: Figure S1. Principal component analysis of soybean PI lines and the Canadian soybean lines (Bastien et al. [14]). The two-dimensional plot (PC1 vs PC2) shows that lines are assigned to two main groups according to their geographical origin. Data not published.

## Abbreviations

DSI: Disease severity index
GBS: Genotyping by sequencing
GWAS: Genome wide-association studies
LD: Linkage disequilibrium
LL: Length of the lesion
QTL: Quantitative trait loci
SNP: Single nucleotide polymorphism
SSR: Sclerotinia stem rot
