# Supplementary material for: Association mapping of QTLs for sclerotinia stem rot resistance in a collection of soybean plant introductions using a genotyping by sequencing (GBS) approach
Source: BMC Plant Biol. 2015 Jan 17;15:5. doi: 10.1186/s12870-014-0408-y (PMC4304118; doi:10.1186/s12870-014-0408-y)
Supplement: Additional file 1: Table S1. — Details of the 101 soybean genotypes used for GWAS. Name of the accession, country of origin, maturity group and mean Sclerotinia stem rot lesion length (LL). [file 12870_2014_408_MOESM1_ESM.docx]

**Table S1. Details of the 101 soybean genotypes used for GWAS. Name of the accession, country of origin, maturity group and mean Sclerotinia stem rot lesion length (LL).**

| Accession | Name | Origin | MG | LL (mm) |
| --- | --- | --- | --- | --- |
| PI 391589B | Hei nung No. 11 | China | I | 13 |
| PI 423949 | saikai 20 | Japan | I | 15 |
| PI 603148 | Oh Won 1 | Korea | I | 16 |
| PI 507352 | Toiku 152 | Japan | II | 16 |
| PI 561345 | Yi tong lu da dou | China | I | 17 |
| PI 196157 | Mituisidaizu | Japan | III | 18 |
| PI 398637 | KAERI-GNT 390-18 | Korea | III | 19 |
| PI 194639 | 741-1 | Sweden | 000 | 21 |
| PI 423871 | Toyosuzu | Japan | I | 21 |
| PI 427141 | Seuhae No. 20 | Korea | I | 22 |
| S19-90 (R) |  | United States | I | 23 |
| PI 358318A | Sapporo Commercial No. 2 | Japan | II | 23 |
| PI 399074 |  | Korea | 0 | 24 |
| PI 467332 | Qing-mo-shi-dou | China | II | 24 |
| PI 372415 A | Hikmok sorip | Korea | II | 25 |
| Kaprio RR (R) |  | Canada | I | 25 |
| PI 189919 | Visuson | France | I | 25 |
| PI 417274 | Sayohime | Japan | II | 26 |
| PI 467312 | Cha-mo-shi-dou (II) | China | II | 27 |
| PI 189861 | Grignon 18 | Germany | I | 27 |
| PI 416930 | Hokkai hadaka | Japan | 0 | 28 |
| PI 248509 B |  | China | II | 28 |
| PI 424148 | KAS 642-2 | Korea | 0 | 28 |
| PI 417268 | Sanryuuiri | Japan | II | 29 |
| PI 437527 | Cel'merezcaja 2 | Ukraine | 0 | 30 |
| PI 358315 C | Tokachi shiro | Japan | I | 30 |
| PI 549066 | Kitanosuzu | Japan | I | 31 |
| PI 567157A | He jiao 11 | China | 0 | 32 |
| PI 593973 | Toyokomachi | Japan | I | 32 |
| PI 416776 | Akita daizu | Japan | I | 32 |
| Karlo RR (R) |  | Canada | I | 33 |
| PI 561331 | Jiao he xiao hei dou | China | I | 34 |
| PI 437764 | VIR 1238 | China | 0 | 35 |
| PI 507353 | Toiku 155 | Japan | II | 35 |
| Majesta (R) |  | Canada | I | 35 |
| PI 548312 | Cayuga | China | I | 36 |
| PI 424242 | KAS 173-5 | Korea | 0 | 36 |
| PR918827 |  | Canada | I | 36 |
| Maple Donovan (R) |  | Canada | 0 | 37 |
| PI 194634 | 737-1 | Sweden | 000 | 37 |
| PI 504502 | Ou yuan tsao shen | Taiwan | I | 37 |
| PI 503336 | Dong Nong 37 | China | 00 | 39 |
| PI 281850 | Wase shiroge | Japan | I | 39 |
| PI 593972 | Suzumaru | Japan | I | 39 |
| PI 458520 |  | China | II | 39 |
| PI 423941 | Orihime | Japan | I | 40 |
| PI 437072 | Amurscaja 21 | Russia | 0 | 40 |
| PI 089001 |  | China | 0 | 41 |
| PI 243547 | Toyo-naga | Japan | 0 | 42 |
| PI 504497 | La po wu | China | II | 47 |
| PI 232996 | No. 113/49 | Germany | 0 | 47 |
| PI 417201 | Nyuuchan | Japan | III | 47 |
| PI 081775 |  | Japan | I | 47 |
| PI 438267 | VIR 5041 | China | 0 | 48 |
| PI 416805 | Asamidori | Japan | I | 50 |
| PI 189896 | Giessner Stamm 63 | Germany | I | 50 |
| PI 467323A | Jiu nong 13 | China | 0 | 50 |
| PI 153316 | Meng Tseu | France | I | 53 |
| PI 423954 | Shirome | Japan | 0 | 53 |
| PI 360850 | Wasekogane | Japan | I | 54 |
| PI 548407 | Sac | Japan | I | 55 |
| PI 578496 | Jin shan pu | China | I | 56 |
| PI 416940 | Hourai | Japan | I | 57 |
| PI 427138 | Choseng 1 | Korea | 0 | 57 |
| PI 427143 | Sipyuk No. 144 | Korea | I | 59 |
| PI 548539 | Comet | Canada | 0 | 59 |
| PI 561285 B | Hei nong 35 | China | I | 60 |
| PI 548404 | Poland Yellow | Canada | 0 | 60 |
| FC 030233 |  | Canada | I | 60 |
| PI 189931 | Jaune De Mandchouria | France | II | 61 |
| PI 153282 | N-23 | Belgium | I | 61 |
| PI 417050 | Kogane daizu | Japan | II | 64 |
| PI 597426 | Gang 81-128-1 | China | 0 | 66 |
| PI 417507 | Bitterhof C | Germany | 0 | 69 |
| PI 189899 | Rouest 104 | France | 0 | 71 |
| PI 549076A | Hong feng No. 3 | China | 0 | 72 |
| PI 578501 | Sui nong No. 4 | China | 0 | 74 |
| PI 132207 | No. D. 14 | Netherlands | 0 | 75 |
| PI 361059B | Crusulea 9/3 | China | 0 | 75 |
| PI 153259 | J-54 | Belgium | 0 | 75 |
| PI 184042 | Nikogri | Yugoslavia | I | 78 |
| OAC Bayfield (S) |  | Canada | 0 | 78 |
| PI 417533 | A425 | Germany | 0 | 78 |
| PI 561367 | Sui nong No. 4 | China | I | 79 |
| PI 291319B |  | China | 0 | 79 |
| Williams 82 (S) |  | United States | II | 80 |
| PI 603375 | Qian guo jian ye he jia dou | China | I | 81 |
| PI 091733 | Grade No. 3 | China | I | 82 |
| PI 561353 | Hei he No. 3 | China | I | 85 |
| PI 417449 | Wase daizu 11 | Japan | 0 | 86 |
| PI 391589A | Hei nung No. 11 | China | I | 87 |
| PI 548380 | Mandarin 507 | China | I | 87 |
| PI 494182 | Suzuhime | Japan | 0 | 88 |
| PI 548354 | Kabott | China | 0 | 89 |
| PI 437654 | Er-hej-jan | China | III | 97 |
| PI 507354 | Tokei 421 | Japan | I | 100 |
| Merit (S) |  | Canada | 0 | 102 |
| Nattosan (S) |  | Canada | 0 | 102 |
| PI 468903 |  | China | I | 111 |
| PI 561284 | Hei nong 34 | China | I | 117 |
| PI 468915 |  | China | II | 124 |
