# Supplementary material for: Association mapping of QTLs for sclerotinia stem rot resistance in a collection of soybean plant introductions using a genotyping by sequencing (GBS) approach
Source: BMC Plant Biol. 2015 Jan 17;15:5. doi: 10.1186/s12870-014-0408-y (PMC4304118; doi:10.1186/s12870-014-0408-y)
Supplement: Additional file 2: Table S2. — Comparison for the mean Sclerotinia stem rot ratings for the field test according to Hoffman et al. [9] and the lesion length for the cotton pad inoculation method (Bastien et al. [12]) of soybean plant introductions and the check cultivar S19-90. [file 12870_2014_408_MOESM2_ESM.docx]

**Table S2. Comparison for the mean Sclerotinia stem rot ratings for the field test according to Hoffman et al. (2002) and the lesion length for the cotton pad inoculation method (Bastien et al., 2012) of soybean plant introductions and the check cultivar S19-90**

|  |  | Hoffman et al. (2002) | Cotton pad method |
| --- | --- | --- | --- |
| Accessions | MG | DSI ^(a)^ | LL^(b)^ |
| PI 391589B | I | 5 | 13 |
| PI 507352 | II | 3 | 16 |
| PI 561345 | I | 13 | 17 |
| PI 196157 | III | 16 | 18 |
| PI 398637 | III | 17 | 19 |
| PI 358318A | II | 10 | 23 |
| PI 189919 | I | 7 | 25 |
| PI 189861 | 0 | 21 | 27 |
| PI 437527 | 0 | 9 | 30 |
| PI 549066 | I | 10 | 31 |
| PI 567157A | 0 | 6 | 32 |
| PI 416776 | I | 10 | 32 |
| PI 561331 | I | 18 | 34 |
| PI 437764 | 0 | 10 | 35 |
| PI 507353 | II | 4 | 35 |
| PI 548312 | I | 6 | 36 |
| PI 504502 | I | 18 | 37 |
| PI 437072 | 0 | 13 | 40 |
| PI 89001 | 0 | 12 | 41 |
| PI 243547 | 0 | 4 | 42 |
| PI 232996 | 0 | 24 | 47 |
| PI 417201 | III | 14 | 47 |
| PI 81775 | I | 14 | 47 |
| PI 438267 | 0 | 18 | 48 |
| PI 416805 | I | 9 | 50 |
| PI 189896 | I | 10 | 50 |
| PI 153316 | I | 11 | 53 |
| PI 548407 | I | 6 | 55 |
| PI 427143 | I | 7 | 59 |
| PI 548539 | 0 | 16 | 59 |
| PI 548404 | 0 | 16 | 60 |
| FC30233 | I | 7 | 60 |
| PI 189931 | II | 17 | 61 |
| PI 153282 | I | 6 | 61 |
| PI 417507 | 0 | 10 | 69 |
| PI 189899 | 0 | 10 | 71 |
| PI 578501 | 0 | 12 | 74 |
| PI 132207 | 0 | 0 | 75 |
| PI 361059B | 0 | 6 | 75 |
| PI 153259 | 0 | 21 | 75 |
| PI 184042 | I | 19 | 78 |
| PI 417533 | 0 | 11 | 78 |
| PI 561367 | I | 6 | 79 |
| PI 291319B | 0 | 9 | 79 |
| PI 91733 | I | 11 | 82 |
| PI 561353 | I | 6 | 85 |
| PI 417449 | 0 | 9 | 86 |
| PI 548380 | I | 19 | 87 |
| PI 548354 | 0 | 18 | 89 |
| PI 561284 | I | 11 | 117 |
| Syngenta S19-90 (R) | I | 10 | 23 |
| Syngenta S19-90 (R) | II | 19 | - |
| Syngenta S19-90 (R) | III | 33 | - |

^a^ DSI = disease severity index that ranged from 0 = all healthy plants with no disease to 100 = all plants killed by disease. The DSI means are based on the disease ratings of 30 plants according to Hoffman et al. (2002).

^b^ Mean lesion length (in millimeters) of soybean cultivars after inoculation with the cotton pad method. Progression of the lesions was assessed 7 d after inoculation at the R1 stage.
