# Supplementary material for: Association mapping of QTLs for sclerotinia stem rot resistance in a collection of soybean plant introductions using a genotyping by sequencing (GBS) approach
Source: BMC Plant Biol. 2015 Jan 17;15:5. doi: 10.1186/s12870-014-0408-y (PMC4304118; doi:10.1186/s12870-014-0408-y)
Supplement: Additional file 3: Figure S1. — Principal component analysis of soybean PI lines and the Canadian soybean lines (Bastien et al. [14]). The two-dimensional plot (PC1 vs PC2) shows that lines are assigned to two main groups according to their geographical origin. Data not published. [file 12870_2014_408_MOESM3_ESM.docx]

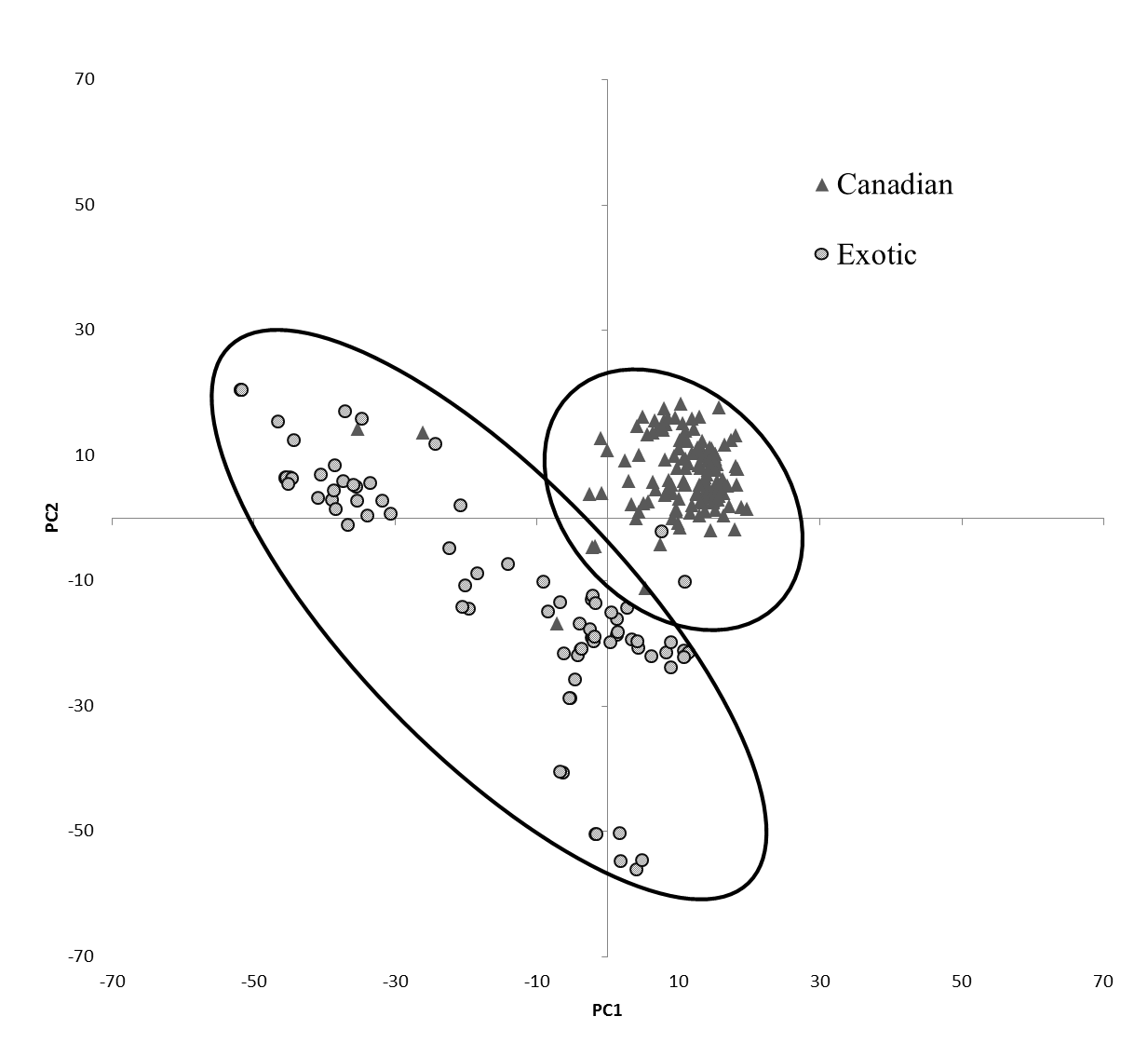


Figure S1. Principal component analysis of 92 soybean PI lines (this study) and 130 Canadian soybean lines from Bastien et al. (2014). The two-dimensional plot (PC1 vs PC2) shows that lines are assigned to two main groups according to their geographical origin.
